# Supplementary material for: Concentration and quantification of Tilapia tilapinevirus from water using a simple iron flocculation coupled with probe-based RT-qPCR
Source: PeerJ. 2022 Apr 18;10:e13157. doi: 10.7717/peerj.13157 (PMC9022640; doi:10.7717/peerj.13157)
Supplement: Supplemental Information 1 [file peerj-10-13157-s001.docx]

**Table S1:** Sample used for evaluation of analytical specificity and sensitivity of the probe-based RT-qPCR method

| Samples | Host | Sample type | RT-qPCR result (Cq) |
| --- | --- | --- | --- |
| Clinically healthy tilapia | NA | RNA | ND |
| NNV-infected tissue | Grouper | RNA | ND |
| ISKNV-infected tissue | Asian sea bass | RNA | ND |
| SDDV-infected tissue | Asian sea bass | RNA | ND |
| *Streptococcus agalactiae* | Nile tilapia | RNA | ND |
| *Streptococcus iniae* | Asian sea bass | RNA | ND |
| *Edwardsiella ictaluri* | Striped catfish | RNA | ND |
| *Edwardsiella tarda* | Nile tilapia | RNA | ND |
| *Flavobacterium columnare* | Asian sea bass | RNA | ND |
| *Francisella orientalis* | Hybrid red tilapia | RNA | ND |
| *Aeromonas hydrophila* | Tilapia | RNA | ND |
| *Aeromonas veronii* | Nile tilapia | RNA | ND |
| *Aeromonas dhakensis* | Hybrid red tilapia | RNA | ND |
| *Aeromonas caviae* | Nile tilapia | RNA | ND |
| *Aeromonas jandaei* | Nile tilapia | RNA | ND |
| *Plesiomonas shigelloides* | Nile tilapia | RNA | ND |
| *Chryseobacterium* sp. | Nile tilapia | RNA | ND |
| *Vogesella* sp. | Nile tilapia | RNA | ND |
| *Vibrio cholerae* | Nile tilapia | RNA | ND |

ND, not detectable
