# Supplementary figures and images for: Concentration and quantification of Tilapia tilapinevirus from water using a simple iron flocculation coupled with probe-based RT-qPCR
Source: PeerJ. 2022 Apr 18;10:e13157. doi: 10.7717/peerj.13157 (PMC9022640; doi:10.7717/peerj.13157)

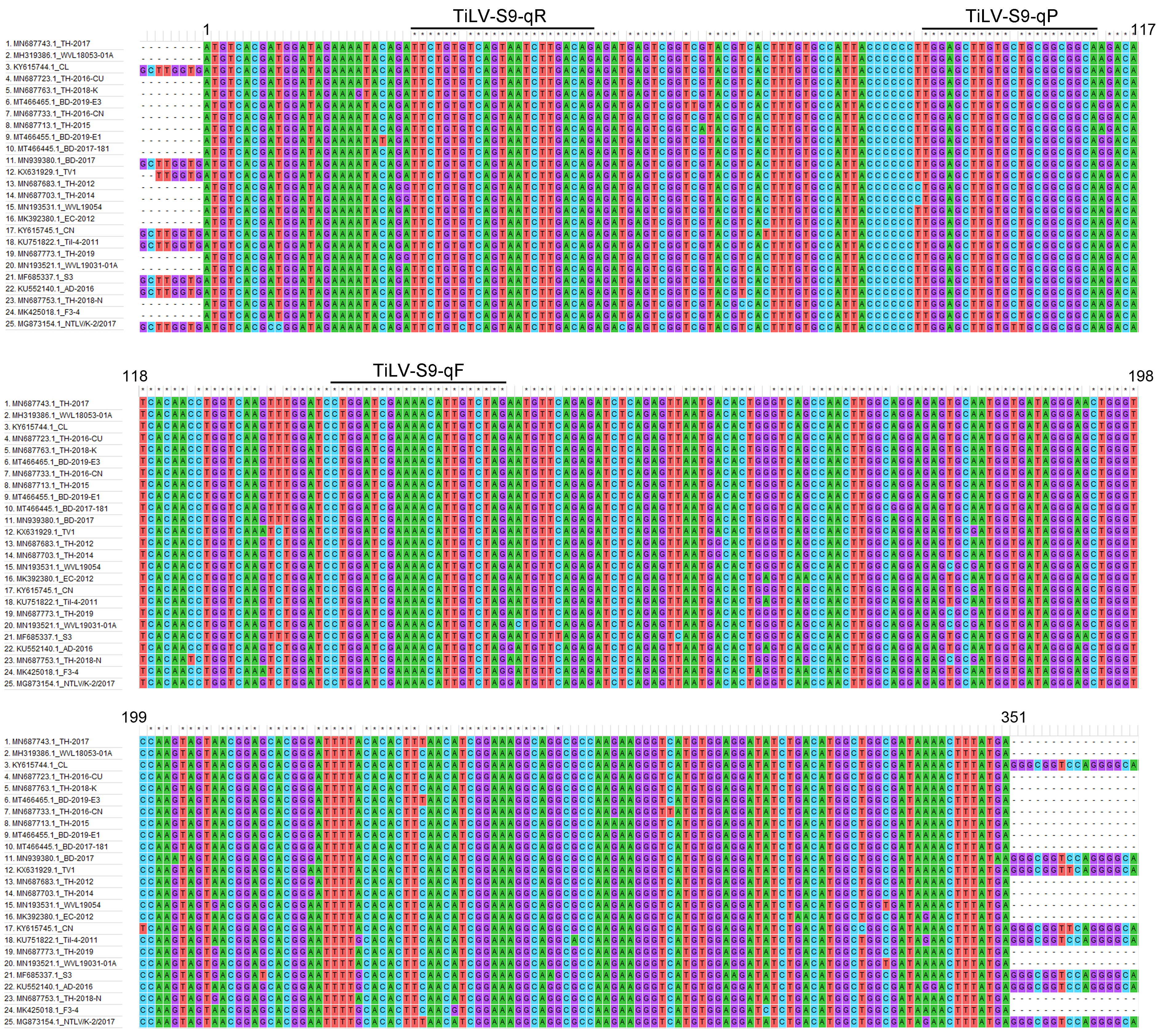

Supplement: Supplemental Information 2 — Accession numbers and viral isolate names of all 25 sequences are shown on the left panel. Position of primers and probe used in the newly developed RT-qPCR assay are marked. Numbers denote nucleotide positions to the putative coding region. [file peerj-10-13157-s002.jpg]
